# Retinopathy of Prematurity and Neurodevelopmental and Quality-of-Life Outcomes at 10 Years of Age

**DOI:** 10.1055/a-2679-1788

**Published:** 2025-08-21

**Authors:** Sudhir Sriram, Elizabeth T. Jensen, Michael E. Msall, Joe X. Yi, Vasyl Zhabotynski, Robert M. Joseph, Karl C.K. Kuban, Jean A. Frazier, Stephen R. Hooper, Hudson P. Santos, Semsa Gogcu, Christiane Dammann, Olaf Dammann, Jeffrey Shenberger, Rebecca C. Fry, Thomas Michael O'Shea, for the ELGAN-ECHO Study Investigators

**Affiliations:** 1 Section of Neonatology, Department of Pediatrics, The University of Chicago, Chicago, Illinois; 2 Department of Epidemiology and Prevention, Wake Forest University School of Medicine, Winston-Salem, North Carolina; 3 Kennedy Research Center on Intellectual and Neurodevelopmental Disabilities, University of Chicago Comer Children's Hospital Section of Developmental and Behavioral Pediatrics, Chicago, Illinois; 4 Department of Pediatrics, University of North Carolina School of Medicine, Chapel Hill, North Carolina; 5 Department of Environmental Sciences and Engineering, Gillings School of Global Public Health, Chapel Hill, North Carolina; 6 Department of Anatomy and Neurobiology, Boston University School of Medicine, Boston, Massachusetts; 7 Division of Pediatric Neurology, Department of Pediatrics, Boston University Medical Center, Boston, Massachusetts; 8 Eunice Kennedy Shriver Center and Department of Psychiatry, University of Massachusetts Chan Medical Center, Worcester, Massachusetts; 9 Department of Health Sciences, School of Medicine, University of North Carolina, Chapel Hill, North Carolina; 10 School of Nursing and Health Studies, University of Miami, Coral Gables, Florida; 11 Department of Pediatrics, Wake Forest University School of Medicine, Winston-Salem, North Carolina; 12 Department of Pediatrics, Tufts University Medical Center, Boston, Massachusetts; 13 Department of Public Health and Community Medicine, Tufts University School of Medicine, Boston, Massachusetts; 14 Department of Neuromedicine and Movement Science, Norwegian University of Science and Technology, Trondheim, Norway; 15 Department of Pediatrics, Connecticut Children's, Hartford, Connecticut

**Keywords:** extremely low gestational age neonates, cognitive function, quality of life, retinopathy of prematurity

## Abstract

**Objective:**

To evaluate, in a cohort of children born extremely preterm, the hypothesis that increasing severity of retinopathy of prematurity (ROP) is associated with less optimal vision, neurodevelopmental outcomes, and parent-reported quality of life.

**Study Design:**

The Extremely Low Gestational Age Newborn study is a multicenter, longitudinal cohort study. Study participants were born before 28 completed weeks of gestation during the years 2002 to 2004 and were enrolled at birth at 14 U.S. hospitals. Based on retinal examinations by ophthalmologists, participants were classified during their initial hospitalization according to the severity of ROP. At 10 years of age, study psychologists evaluated participants' cognitive abilities, academic achievement, and behaviors indicative of autism spectrum disorder. Participants were classified with regard to gross motor function, anxiety, depression, and quality of life based on parents' responses on standardized questionnaires.

**Results:**

After adjustment for confounders, increased severity of ROP was associated with increased severity of vision/eye problems, worse scores on math achievement tests, as well as higher prevalence of anxiety and lower quality of life as reported by the parent when the child was 10 years old. A history of blindness in one or both eyes was associated with these same outcomes, as well as worse scores on assessments of cognitive function, reading ability, and social responsiveness.

**Conclusion:**

Among extremely preterm children, severe ROP and severe eye or vision problems are associated with adverse neurodevelopmental outcomes and lower quality of life.

**Key Points:**

- Severe retinopathy of prematurity in extremely low gestational age neonates (ELGANs) is likely to have adverse outcomes at 10 years of age.
- Severe ROP in ELGANs is associated with parent-reported lower quality of life at 10 years.
- Blindness in one or both eyes following ROP is associated with multiple adverse outcomes at 10 years.

Survival of extremely low gestational age neonates (ELGANs <28 weeks' gestational age) has improved over the past three decades. 1 2 3 4 Survivors are at high risk for retinopathy of prematurity (ROP), with a prevalence of 10 to 33%. 2 5 In most cases, ROP resolves without serious visual sequelae; however, severe ROP is associated with worse cognitive and motor abilities in early childhood, 5 6 7 8 even after adjusting for neonatal brain injury. Further, since the brain and eye share embryonic origins, risk factors associated with ROP, such as oxidative stress and inflammation, might have harmful effects on the development of the central nervous system. 9 10 11 Nonetheless, few studies of neurodevelopmental outcomes among children with a history of ROP have followed individuals into older childhood. 12 13 14

The follow-up study of Cryotherapy for Retinopathy of Prematurity (CRYO-ROP) demonstrated that very low birth weight infants who develop threshold ROP are at increased risk of adverse vision outcomes and functional limitations in self-care, continence, mobility, communication, and social cognition skills at 5 years of age. 6 At 8 years of age, CRYO-ROP study participants with unfavorable vision status had worse developmental, educational, and social skills than those who had favorable vision status. 15 Furthermore, in a cohort of extremely low birth weight infants born in Victoria, Australia between 1991 and 1992 ( *n* = 180), participants who previously had severe ROP exhibited more impairments at 17 to 18 years of age than peers without severe ROP on measures of visual processing, visual–motor integration, visual learning, intelligence quotient (IQ), and academic achievement skills in reading, spelling, and math. After controlling for perinatal risk factors, only visual acuity scores remained significant between the groups. 12

These studies describing the relationship between severe ROP and neurodevelopmental outcomes in school-aged children did not include children born within the past two decades. Recently, a secondary analysis of data collected from the South Korean National Health Insurance Service database throughout the 10-year follow-up found a higher prevalence of developmental delay in children with severe ROP compared with those with mild ROP, although this study was based on registry data unadjusted for neonatal morbidities (mechanical ventilation, necrotizing enterocolitis, bronchopulmonary dysplasia, periventricular leukomalacia, and severe intraventricular hemorrhage [IVH]), which also may have been contributory to neurodevelopmental impairments. 13

To increase the understanding of associations between severe ROP and neurocognitive, neurobehavioral, and quality-of-life (QoL) outcomes at school age, and to counsel the parents of ELGANs, we analyzed the data from the ELGAN Study of neonates born before 28 weeks of gestation, and prospectively followed to 10 years of age. We hypothesized that extremely preterm infants with severe ROP would have less favorable neurocognitive, neurobehavioral, and QoL outcomes in middle childhood as compared with ELGAN participants without severe ROP.

## Materials and Methods

### Participants

**Fig. 1:**
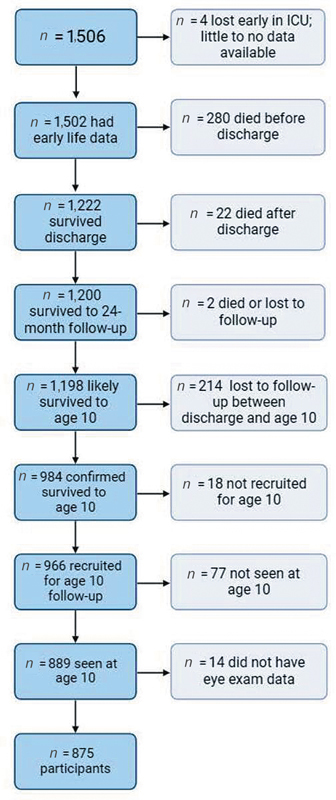
Flow chart.

The ELGAN study is a multicenter, prospective, observational study of the risk of structural and functional neurological disorders in extremely preterm infants. 16 17 A total of 1,506 infants born before 28 weeks of completed gestation (median = 26; range 23–27) were enrolled at 14 hospitals in five states in the United States within a few days of birth during the years 2002 to 2004; 1,222 were discharged alive from neonatal intensive care units (NICUs) and 1,198 survived to 10 years. Of those who survived, 309 (26%) children did not return for follow-up ( *n* = 214 lost for follow-up between discharge and age 10; *n* = 18 not recruited for follow-up at age 10; *n* = 77 not seen at age 10; Fig. 1 ). A total of 889 (74%) returned for follow-up. After excluding 14 children who did not have retinal examination data to classify their ROP status, our final sample was 875 children (73% of surviving members of the cohort; Fig. 1 ).

### Perinatal Data

Within a few days of the birth of study participants, mothers were interviewed by a research coordinator who collected information on mother's age, eligibility for government-subsidized health insurance, years of formal education completed, and marital status. Research coordinators reviewed mothers' medical records to obtain information about pregnancy complications and medical treatments and reviewed neonatal records to collect information about neonatal illnesses and treatments. As described in detail elsewhere, cranial ultrasounds were obtained as a component of standard care, using the anterior fontanelle as the sonographic window. Scans were performed with digital high-frequency transducers (7.5 and 10 megahertz). All scans included the 6 standard quasi-coronal views and 5 sagittal views; mastoid window views of the cerebellum were not routinely obtained. To identify cerebral white matter abnormalities and persistent ventricular enlargement, we used scans obtained between the 15 ^th^ postnatal day and 40 weeks' postmenstrual age. All scans were read by two independent radiologists unaware of clinical information. A third reader, unaware of the initial two reads, was used as a tiebreaker when the initial two readers differed in their recognition of IVH, echolucent (hypoechoic) parenchymal lesions, or moderate to severe ventriculomegaly. For the current study, we defined white matter abnormality as the presence of either parenchymal echolucency (hypoechoic abnormality), parenchymal echodensity (hyperechoic abnormality), or moderate to severe ventriculomegaly on a “late” scan (performed after the first two postnatal weeks). 18

### Retinopathy of Prematurity

Retinal examinations were performed by ophthalmologists experienced in ROP screening at each NICU where participants received care. Participating ophthalmologists helped to prepare a manual and data collection form, and then participated in efforts to minimize observer variability. Definitions of terms were those accepted by the International Committee for Classification of Retinopathy of Prematurity and the Committee for the Classification of Retinopathy of Prematurity (1984). 19 20 21 In keeping with these guidelines, the first ophthalmologic examination was performed between the 31 ^st^ and 33 ^rd^ postmenstrual week. Follow-up exams were performed as clinically indicated until normal vascularization began in zone III. For the current analyses, severe ROP was defined as ROP stage 3, 4, 5 and was stratified into those not requiring and those requiring surgery; less-severe ROP was defined as ROP stage 1, 2 and not requiring surgery. ELGAN participants without ROP served as the comparison group.

### 10-Year Follow-up Visit

Assessments were selected to provide the most comprehensive information about cognitive functions and selected academic skills. While the child was tested, the parent or caregiver completed a general health questionnaire (medical diagnoses and receipts of medications), as well as questionnaires about functional communication, social–emotional and adaptive behaviors, and QoL. Evaluations for 10-year follow-up visit were scheduled between April 2012 and August 2014.

### Eye Disorders at 10 Years

At the time of the 10-year follow-up visit, a parent or guardian reported whether the child had the following eye disorders: requirement for corrective lenses, blindness in one or both eyes, and strabismus requiring surgery or not. Based on these data, study participants were classified according to the most severe vision or eye problem using the following hierarchy: none, corrective lenses, strabismus not requiring surgery, strabismus requiring surgery, blind in one eye, or blind in both eyes.

### Cognitive Function

To evaluate cognitive functioning at 10 years of age, we used the school-age Differential Ability Scales-II (DAS-II) 22 and the “Developmental NEuroPSYchological Assessment” (NEPSY-II). 23 Participants who were not able to obtain basal score on any given test because of severe motor impairment were assigned the lowest score for the given test. We employed latent profile analysis to identify subgroups of ELGANs with similar profiles on nine measures of verbal and nonverbal IQ (DAS-II Verbal and Nonverbal Reasoning scales), working memory (DAS-II Recall of Digits Backward, Recall Sequential Order), concept generation and mental flexibility (NEPSY-II Animal Sorting), auditory attention and set switching (NEPSY-II Auditory Attention, Response Set), and simple inhibition and inhibition shifting (NEPSY-II Inhibition and Inhibition Switching). Using this approach, four neurocognitive profiles were identified: (1) “normal”—mean IQ and executive function scores within the normal range (<0.5 standard deviation [SD]) on all measures; (2) “low-normal”—mean IQ and executive function scores ranged from 0.5 to 1.5 SDs below the mean in the normative sample; (3) “moderately impaired”—mean IQ and executive function scores between 1.5 and 2.5 SDs below the mean in the normative sample; and 4) “severely impaired”—mean IQ and executive function scores between 2.5 and 4 SDs below the mean in the normative sample. 24 Academic achievement was evaluated using the Wechsler Individual Achievement Test-III (WIAT-III) Word Reading and Numerical Operations subtests. 25

### Gross Motor Function

Gross motor function was assessed with the Gross Motor Function Classification System, an ordinal scale that ranges from I to V, with higher levels indicative of greater functional limitations. 26

### Epilepsy

Epilepsy was identified using a two-stage screening process that included administration of a brief screening questionnaire to the parents, followed by an open-ended interview conducted by an epileptologist. 27 28 A diagnosis of epilepsy required the consensus of two epileptologists and was defined as two or more seizures after the neonatal period that were not provoked by fever, trauma, or infection of the central nervous system.

### Autism Assessment and Social Responsiveness Scales

Parents completed the Social Communication Questionnaire, and if the score was greater than 11, they were asked to complete the Autism Diagnostic Interview-Revised (ADIR). Children who met the ADIR criteria for autism spectrum disorder (ASD) were evaluated with the Autism Diagnostic Observation Schedule 2 (ADOS2). All children who met standardized criteria for ASD on both ADIR and ADOS2 were classified as having ASD. We used the Social Responsive Scale (SRS), completed by the parent, to assess a constellation of social challenges in social awareness, social cognition, social communication, social motivation and autistic mannerisms (stereotypes and restricted interests), behaviors typically observed in children with an ASD. As previously described, SRS was modeled as a binary outcome (≤65 or >65). 29 Scores > 65 indicate more impaired social responsiveness.

### Attention Deficit Hyperactivity Disorder

The parent or caregiver completed the Child Symptom Inventory (CSI-4) Parent Check List, 30 and the child's current teacher completed the teacher CSI-4 checklist. To parallel clinical decision making, three contexts in which attention deficit hyperactivity disorder (ADHD) symptoms could manifest were considered. Two of the contexts were taken from the CSI-4 norm-based cutoffs for the parent and teacher reports of ADHD symptoms, and the third context was based on the parent's indication at interview of the child being diagnosed previously to have ADHD by a clinician. Participants were included in the ADHD symptom group if they met any 2 or 3 criteria. 31 32 33

### Anxiety and Depression

For identification of anxiety disorders and depressive disorders, parents completed the CSI-4 parent checklist and teachers completed CSI-4 teacher checklist. 31 The forms asked for details about generalized anxiety, social phobia, depressive disorder, or dysthymic disorder. For this analysis, we defined parent-reported anxiety as an average T-score > 60 across three CSI-4 scales: general anxiety, separation anxiety, social phobia; and parent-reported depression as an average T-score > 60 across two CSI-4 scales: dysthymia and major depression. Teacher-reported anxiety and depression were defined analogously, except that only general anxiety and social phobia were included in the definition of anxiety because the teacher-reported CSI-4 scale does not include items on which the T-score for separation anxiety is based.

### Pediatric Quality-of-Life Inventory

For the QoL measure, a parent or a caregiver completed the PedsQL 4.0 generic core scales at 10-year follow-up. 34 35 The 23-item PedsQL was designed to measure core dimensions of QoL in areas of physical (8 items), emotional (5 items), social (5 items), and school (5 items) with each item scored on a five-point Likert scale. Each dimension was summed and linearly transformed to a 0 to 100 scale with higher scales indicating better QoL. In our analyses, we dichotomized the total PedsQL using a cutoff of <70, which is 2 SDs below the mean in the reference sample.

### Data Analysis

To analyze associations between severity of ROP and outcomes at 10 years of age, we used chi-square tests for univariable analysis and logistic regression models for multivariable analyses. The strength of association was estimated as odds ratios and adjusted odds ratios (aOR) and 95% confidence intervals (CI), with individuals without ROP as the referent group. Goodness of fit was assessed using a Pearson's chi-square where the null hypothesis is that the fitted model is correct.

**Fig. 2:**
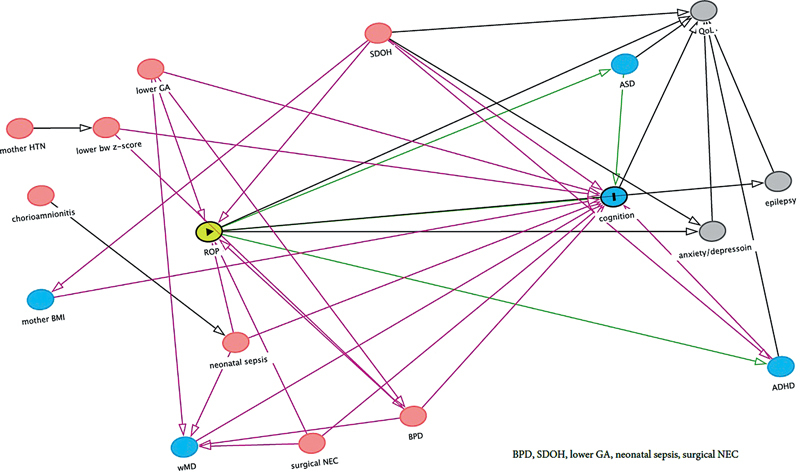
Directed acyclic graph.

We created directed acyclic graphs 36 to identify the minimally sufficient adjustment sets of variables to include in multivariable regression models to close noncausal, biasing pathways ( Fig. 2 ). Multivariable analyses adjusted for maternal age, eligibility for government-subsidized health insurance, number of years of formal education completed by the mother at the time of the study participant's birth, marital status (married vs. unmarried), duration of mechanical ventilation, gestational age, sepsis, sex, and surgical necrotizing enterocolitis. In a sensitivity analysis, we adjusted for the set of variables listed above plus maternal fever during pregnancy. In sensitivity analyses, we applied parametric multiple imputation to evaluate the potential for bias resulting from informative missingness due to sample attrition. Missing values were imputed using the adjustment covariates, and 10 iterations of imputed datasets were analyzed; final odds ratios represent the mean odds ratios of the 10 iterations.

## Results

### Study Participants

Children who were evaluated at 10 years of age ( *n* = 889), as compared with children who participated in the study, but not the study sample ( *n* = 309) were similar in maternal variables except that the study sample had slightly lower proportions of participants born to mothers with high school education or less, Medicaid eligibility, and Black race. Infant variables were similar for those evaluated at 10 years as compared with children who participated in the study, but not the study sample except multiple gestations and chronic lung disease were higher in children who were in the study sample( Table 1 ). Of the 875 children included in the present analysis, 16% ( *n* = 138) had severe ROP (stages 3–5) requiring surgery; 15% ( *n* = 129) had severe ROP not requiring surgery; 44% ( *n* = 385) had less severe ROP (stages 1 and 2) not requiring surgery; and 25% ( *n* = 223) did not have ROP ( Table 2 ). Table 2 summarizes characteristics of mothers and neonates for study participants grouped according to severity of ROP.

**Table 1:** Comparison of ELGAN Study participants who were discharged alive from the neonatal intensive care unit and those evaluated at 10 years of age

Characteristics		Alive at 10 y but not in sample ( *n* = 309)	Age 10 study sample ( *n* = 889)
Maternal age (y)	<21	55 (18%)	115 (13%)
	21–35	208 (67%)	594 (67%)
	>35	46 (15%)	180 (20%)
Unmarried mother		160(52%)	353 (40%)
Maternal education (y)	≤12	151 (52%)	355 (41%)
	>12 to <16	68 (24%)	202 (23%)
	≥16	70 (24%)	306 (36%)
Mother covered by Medicaid or another state-supported medical insurance		157 (53%)	307 (35%)
Race	Race other than White or Black	53 (18%)	98 (11%)
	Black	95 (32%)	227 (26%)
	White	152 (51%)	554 (63%)
Hispanic	Yes	61 (20%)	86 (10%)
	No	245 (80%)	800 (90%)
Mother's prepregnancy body mass index (kg/m ^2^ )	<18.5	22 (8%)	68 (8%)
	18.5– < 30	214 (74%)	595 (69%)
	≥30	54 (19%)	194 (23%)
Cesarean delivery		205 (66%)	590 (66%)
Sex	Male	166 (54%)	455 (51%)
	Female	143 (46%)	434 (49%)
Multiple gestation		69 (24%)	293 (35%)
Gestational age (wk)	23–24	58 (19%)	187 (21%)
	25–26	153 (50%)	400 (45%)
	27	98 (32%)	302 (34%)
Birth weight (g)	≤750	104 (34%)	332 (37%)
	750–1,000	138 (45%)	382 (43%)
	>1,000	67 (22%)	175 (20%)
Birth weight Z-score < −2		9 (3%)	53 (6%)
Average daily weight gain during neonatal intensive care	Lowest quartile	87 (28%)	214 (24%)
	Highest quartile	68 (22%)	231 (26%)
Bacteremia		85 (28%)	256 (29%)
White matter injury on neonatal ultrasound a		54 (18%)	188 (21%)
Severe retinopathy of prematurity b		38 (13%)	118 (14%)
No necrotizing enterocolitis		246 (80%)	689 (78%)
Medical necrotizing enterocolitis		46 (15%)	138 (16%)
Surgical necrotizing enterocolitis		11 (4%)	33 (4%)
Spontaneous intestinal perforation		6 (2%)	29 (3%)
Chronic lung disease c		137 (45%)	461 (52%)

Abbreviation: NICU, neonatal intensive care unit.

Note: Data are number of children (percent of group).

a White matter injury was defined as one or more of the following findings, with agreement by two readers, on cranial ultrasound: persistent ventricular enlargement beyond the first month of life, echodensity in the periventricular white matter, echolucency in the periventricular white matter.

b Severe retinopathy of prematurity defined as meeting criteria for the Early Treatment of Retinopathy of Prematurity Trial.

c Defined as receipt of supplemental oxygen at 36 weeks' postmenstrual age; missing for *n* = 7 in study sample and *n* = 3 not in sample.

**Table 2:** Maternal and neonatal characteristics as a function of severity of retinopathy of prematurity

		Severity of retinopathy of prematurity			
Characteristics		No ROP ( *n* = 223)	Less severe ROP; no surgery ( *n* = 385)	More severe ROP no surgery ( *n* = 129)	ROP requiring surgery ( *n* = 138)
Maternal age (y)	<21	20 (9%)	58 (15%)	12 (9%)	22 (16%)
	21–35	154 (69%)	248 (64%)	96 (74%)	88 (64%)
	>35	49 (22%)	79 (21%)	21 (16%)	28 (20%)
Unmarried mother		91 (41%)	161 (42%)	54 (42%)	40 (29%)
Maternal education (y)	≤12	78 (36%)	165 (44%)	49 (39%)	58 (43%)
	>12– < 16	47 (22%)	82 (22%)	31 (25%)	39 (29%)
	≥16	92 (42%)	126 (34%)	45 (36%)	37 (28%)
Mother covered by Medicaid or another state-supported medical insurance		71 (32%)	142 (37%)	48 (38%)	40 (30%)
Race	Asian, Native American, or mixed race	22 (10%)	45 (12%)	15 (12%)	14 (10%)
	Black	56 (25%)	95 (25%)	41 (32%)	31 (23%)
	White	144 (65%)	242 (63%)	71 (56%)	89 (66%)
Hispanic	No	209 (94%)	348 (91%)	112 (88%)	118 (86%)
	Yes	14 (6%)	35 (9%)	16 (13%)	20 (15%)
Mother's prepregnancy body mass index (kg/m ^2^ )	<18.5	19 (9%)	33 (9%)	12 (10%)	4 (3%)
	18.5 to <30	148 (68%)	262 (70.8%)	78 (64%)	98 (74%)
	≥30	52 (24%)	75 (20.3%)	32 (26%)	31 (23%)
Clinical chorioamnionitis		111 (55%)	189 (52.5%)	56 (48%)	78 (60%)
Indication for delivery	Preterm labor	102 (46%)	182 (47.3%)	59 (46%)	62 (45%)
	Premature rupture of membranes	59 (27%)	77 (20.0%)	25 (19%)	26 (19%)
	Preeclampsia	25 (11%)	44 (11.4%)	27 (21%)	16 (12%)
	Placental abruption	19 (9%)	46 (11.9%)	11 (9%)	15 (11%)
	Cervical insufficiency	12 (5%)	18 (4.7%)	4 (3%)	10 (7%)
	Fetal indication	6 (3%)	18 (4.7%)	3 (2%)	9 (7%)
Cesarean delivery		143 (64%)	259 (67%)	88 (68%)	91 (66%)
Sex	Female	103 (46%)	192 (50%)	60 (47%)	71 (51%)
	Male	120 (54%)	193 (50%)	69 (54%)	67 (49%)
Multiple gestation		79 (38%)	127 (35%)	42 (35%)	43 (32%)
Gestational age (wk)	23–24	10 (5%)	74 (19%)	35 (27%)	68 (49%)
	25–26	90 (40%)	178 (46%)	71 (55%)	58 (42%)
	27	123 (55%)	133 (35%)	23 (18%)	12 (9%)
Birth weight (g)	≤750	33 (15%)	138 (36%)	69 (54%)	91 (66%)
	750–1,000	104 (47%)	175 (46%)	53 (41%)	42 (30%)
	>1,000	86 (39%)	72 (19%)	7 (5%)	5 (4%)
Birth weight z-score	< −2	6 (3%)	19 (5%)	13 (10%)	15 (11%)
	≥ −2, < −1	13 (6%)	57 (15%)	25 (19%)	24 (17%)
	> = −1	204 (92%)	309 (80%)	91 (71%)	99 (72%)
White matter injury on neonatal ultrasound a		45 (20%)	74 (19%)	29 (23%)	38 (28%)
Definite sepsis		57 (26%)	102 (27%)	50 (39%)	48 (35%)
Presumed sepsis		59 (27%)	136 (35%)	48 (37%)	46 (33%)
No sepsis		107 (48%)	147 (38%)	31 (24%)	44 (32%)
No necrotizing enterocolitis		193 (87%)	305 (79%)	86 (67%)	92 (67%)
Necrotizing enterocolitis, medical		24 (11%)	57 (15%)	29 (23%)	27 (20%)
Necrotizing enterocolitis, surgical		5 (2%)	11 (3%)	5 (3.9%)	12 (8.7%)
Spontaneous intestinal perforation		1 (0.4%)	12 (3%)	9 (7.0%)	7 (5.1%)
Chronic lung disease b		60 (27%)	194 (51%)	91 (70.5%)	114 (82.6%)
SNAP score	<20	145 (67%)	206 (55%)	54 (42.2%)	44 (32.1%)
	20–29	45 (21%)	85 (23%)	38 (29.7%)	44 (32.1%)
	30+	28 (13%)	86 (23%)	36 (28.1%)	49 (35.8%)

Abbreviations: ROP, retinopathy of prematurity; SNAP, Score for Neonatal Acute Physiology.

Note: Data are number of study participants, with column totals in parentheses.

a White matter injury was defined as one or more of the following findings, with agreement by two readers, on cranial ultrasound:

Persistent ventricular enlargement beyond the first month of life, echodensity in the periventricular white matter.

Echolucency in the periventricular white matter.

b Chronic lung disease was defined as receipt of supplemental oxygen at 36 weeks' postmenstrual age.

### Outcomes at 10 Years with and without Retinopathy of Prematurity

We compared children with and without ROP for vision outcomes (i.e., need for eyeglasses, strabismus and strabismus surgery, and blindness in one or both eyes), neurocognitive outcomes (i.e., moderate–severe cognitive impairment, math scores, ASD, ADHD, parent-reported anxiety), motor impairment, and QoL in children. No differences were found in eye problems such as strabismus, corrective lenses, among the children with and without ROP, except that blindness was more frequent in children with ROP 58/652 (9%) versus 4/223 (2%) in no ROP ( Table 3 ).

**Table 3:** Outcomes at 10 years of age for children with and without retinopathy of prematurity

Outcomes	None ( *n* = 223)	ROP ( *n* = 652)	Unadjusted odds ratio (95% confidence interval)	Adjusted odds ratio (95% confidence interval) a
No eye or vision problem at 10 y of age	145 (65%)	289 (44%)	0.4 (0.3, 0.6)	0.6 (0.4, 0.9)
Eyeglasses	70 (31%)	309 (47%)	2.0 (1.4, 2.7)	1.4 (0.9, 1.9)
Strabismus, no surgery	17 (7%)	116 (18%)	2.6 (1.5, 4.5)	1.7 (0.9, 3.0)
Strabismus surgery	9 (4%)	72 (11%)	3.0 (1.5, 6.0)	1.7 (0.8, 3.8)
Blind in one eye	0	28 (4%)	b	b
Blind in both eyes	4 (2%)	30 (5%)	2.6 (0.9, 7.6)	1.0 (0.3, 3.3)
Normal cognitive function	91 (41%)	203 (32%)		
Low normal cognitive function	90 (41%)	264 (41%)		
Moderately impaired cognitive function	32 (15%)	113 (18%)		
Severely impaired cognitive function	8 (4%)	60 (9%)	1.7 (1.1, 2.5) c	0.9 (0.6, 1.4) c
Word reading < 70	18 (8%)	78 (12%)	1.5 (0.9, 2.7)	0.8 (0.4, 1.5)
Numerical operations < 70	22 (10%)	112 (18%)	1.9 (1.2, 3.1)	1.1 (0.6, 1.9)
Autism spectrum disorder	7 (3%)	53 (8%)	2.8 (1.3, 6.2)	1.5 (0.6, 3.6)
SRS ≥ 65 (children without ASD and with IQ ≥ 70)	22 (10%)	64 (11%)	1.0 (0.6, 1.7)	1.1 (0.6, 1.9)
ADHD	30 (14%)	121 (19%)	1.5 (1.0, 2.3)	1.1 (0.7, 1.8)
Anxiety-parent report	12 (5%)	78 (12%)	2.4 (1.3, 4.6)	2.0 (1.0, 3.9)
Depression-parent report	6 (3%)	29 (5%)	1.7 (0.7, 4.2)	1.6 (0.6, 4.1)
Anxiety-teacher report	10 (6%)	40 (8%)	1.4 (0.7, 2.9)	1.1 (0.5, 2.4)
Depression-teacher report	9 (6%)	15 (3%)	0.5 (0.2, 1.3)	0.5 (0.2, 1.3)
Epilepsy	19 (9%)	47 (7%)	0.8 (0.5, 1.5)	0.5 (0.3, 1.0)
GMFCS ≥ 3	9 (4%)	63 (10%)	2.6 (1.3, 5.3)	1.2 (0.6, 2.7)
Peds quality of life < 70	38 (17%)	174 (27%)	1.8 (1.2, 2.6)	1.2 (0.8, 1.8)

Abbreviations: ADHD, attention deficit hyperactivity disorder; ASD, autism spectrum disorder; GMFCS, Gross Motor Function Classification System; IQ, intelligence quotient; ROP, retinopathy of prematurity; SRS, Social Responsiveness Scale (scores ≥ 65 indicate more impaired social responsiveness).

a Multivariable analyses adjusted for maternal age, eligibility for government-subsidized health insurance, maternal years of formal education, marital status (married vs. unmarried), duration of mechanical ventilation, gestational age, any early or late sepsis, and surgical necrotizing enterocolitis (Penrose drain, exploratory laparotomy, or both).

b Relative risk cannot be estimated due to inadequate sample size.

c Odds ratio for outcome of severely or moderately impaired cognitive function (normal or low normal cognitive function as referent).

### Retinopathy of Prematurity Severity and Outcomes at 10 Years of Age

Table 4 summarizes the prevalences of cognitive, behavioral, neurological, and QoL outcomes as a function of ROP severity, and Table 5 summarizes associations between ROP severity and outcomes at 10 years. Notably, some children without ROP, nonetheless developed eye problems, such as strabismus (6%) and blindness (2%). In general, as compared with individuals without ROP, those with less severe ROP had similar prevalences of eye problems; those who required surgery for ROP had the highest prevalence of problems; and those with more severe ROP who did not require surgery had prevalences intermediate between those with no ROP and those who required surgery. With participants who had no ROP as the referent group, adverse outcomes that were most strongly associated with ROP requiring surgery (odds ratios ≥ 2) were low scores on numerical operations, parent-reported anxiety, motor impairment, and QoL. For the exposure of more severe ROP, which did not require surgery, the strongest associations were with ASD (aOR: 2.1; 95% CI: 0.8, 5.9). For less severe ROP, the only association with aOR > 1.5 was with parent-reported anxiety. Similar findings were obtained in a sensitivity analysis in which maternal fever was added as a covariate and a sensitivity analysis restricted to participants without ultrasound-identified white matter disease (WMD).

**Table 4:** Outcomes at 10 years of age as a function of retinopathy of prematurity severity

Outcomes	Severity of retinopathy of prematurity			
	No ROP ( *n* = 223)	Less severe ROP; no surgery ( *n* = 385)	More severe ROP no surgery ( *n* = 129)	ROP requiring surgery ( *n* = 138)
No eye or vision problem at 10 y of age	149 (67%)	242 (63%)	58 (45%)	15 (11%)
Eyeglasses	50 (22%)	89 (23%)	33 (26%)	52 (38%)
Strabismus	13 (6%)	25 (7%)	12 (9%)	18 (13%)
Strabismus surgery	7 (3%)	21 (6%)	11 (9%)	18 (13%)
Blind in one eye	0	6 (2%)	9 (7%)	13 (9%)
Blind in both eyes	4 (2%)	2 (1%)	6 (5%)	22 (16%)
Normal cognitive function	91 (41%)	132 (35%)	38 (30%)	33 (25%)
Low normal cognitive function	90 (41%)	157 (41%)	56 (44%)	51 (38%)
Moderately impaired cognitive function	32 (15%)	68 (18%)	19 (15%)	26 (20%)
Severely impaired cognitive function	8 (4%)	22 (6%)	15 (12%)	23 (17%)
Word reading < 70	18 (8%)	33 (9%)	19 (15%)	26 (20%)
Numerical operations < 70	22 (10%)	54 (14%)	24 (19%)	34 (26%)
Autism spectrum disorder	7 (3%)	26 (7%)	14 (11%)	13 (11%)
SRS ≥ 65 (children without ASD and with IQ ≥ 70)	22 (10%)	32 (8%)	16 (12%)	16 (12%)
ADHD	32 (14%)	78 (20%)	24 (17%)	32 (19%)
Anxiety-parent report	12 (5%)	44 (12%)	11 (9%)	23 (18%)
Depression-parent report	6 (3%)	17 (5%)	5 (4%)	7 (5%)
Anxiety-teacher report	10 (6%)	27 (10%)	7 (8%)	6 (6%)
Depression-teacher report	9 (6%)	12 (4%)	3 (3%)	0
Epilepsy	19 (9%)	23 (6%)	7 (5%)	17 (12%)
GMFCS≥ 3	8 (4%)	12 (3%)	6 (5%)	18 (13%)
Peds quality of life < 70	38 (17%)	84 (22%)	43 (34%)	47 (36%)

Abbreviations: ADHD, attention deficit hyperactivity disorder; ASD, autism spectrum disorder; GMFCS, Gross Motor Function Classification System; IQ, intelligence quotient; ROP, retinopathy of prematurity; SRS, Social Responsiveness Scale (scores ≥ 65 indicate more impaired social responsiveness).

Note: Data are number of study participants, with column percentages in parentheses.

**Table 5:** Associations between severity of retinopathy of prematurity and outcomes at 10 years of age

	Sample size of model	Unadjusted OR			Adjusted OR a		
		Less severe ROP no surgery vs. no ROP	More severe ROP no surgery vs. no ROP	ROP requiring surgery vs. no ROP	Less severe ROP no surgery vs. no ROP	More severe ROP no surgery vs. no ROP	ROP requiring surgery vs. no ROP
Cognitive impairment	861	1.4 (0.9, 2.1)	1.6 (01.0, 2.8)	2.64 (1.6, 4.3)	1.0 (0.7, 1.7)	0.9 (0.5, 1.7)	1.6 (0.9, 2.8)
Word Reading < 70	845	1.1 (0.6, 2.0)	2.0 (1.0, 4.0)	2.8 (1.5, 5.3)	0.7 (0.4, 1.4)	1.0 (0.5, 2.2)	1.5 (0.7, 3.3)
Numerical operations < 70	855	1.5 (0.9, 2.5)	2.1 (1.1, 4.0)	3.12(1.8, 5.7)	1.2 (0.7, 2.1)	1.4 (0.7, 2.9)	2.3 (1.1, 4.6)
Autism spectrum disorder	843	2.2 (0.9, 5.2)	3.9 (1.5, 9.9)	3.6 (1.4, 9.3)	1.5 (0.6, 3.7)	2.1 (0.8, 5.9)	1.7 (0.6, 5.0)
SRS ≥ 65 (children without ASD and with IQ ≥ 70)	817	0.8 (0.5, 1.5)	1.39 (0.7, 2.6)	1.2 (0.6, 2.4)	0.8 (0.4, 1.5)	1.37 (0.64, 2.95)	1.5 (0.7, 3.5)
ADHD	860	1.6 (1.0, 2.5)	1.3 (0.7, 2.3)	1.5 (0.8, 2.7)	1.3 (0.8, 2.1)	0.9 (0.4, 1.7)	1.0 (0.5, 2.0)
Anxiety-parent report	857	2.3 (1.2, 4.4)	1.7 (0.7, 3.9)	3.7 (1.8, 7.8)	2.3 (1.1, 4.5)	1.6 (0.6, 3.9)	4.1 (1.7, 9.6)
Depression-parent report	857	1.7 (0.7, 4.3)	1.5 (0.5, 5.0)	2.0 (0.7, 6.2)	1.6 (0.6, 4.3)	1.4 (0.4, 5.3)	2.0 (0.5, 7.1)
Anxiety-teacher report	631	1.6 (0.8, 3.4)	1.3 (0.5, 3.5)	1.0 (0.3, 2.7)	1.4 (0.6, 3.0)	1.1 (0.4, 3.2)	0.7 (0.2, 2.2)
Depression-teacher report	628	0.7 (0.3, 1.8)	0.6 (0.2, 2.2)	b	0.8 (0.3, 2.2)	0.7 (0.2, 3.0)	b
Epilepsy	874	0.7 (0.4, 1.3)	0.6 (0.3, 1.5)	1.5 (0.8, 3.0)	0.5 (0.3, 1.0)	0.4 (0.1, 1.0)	1.0 (0.4, 2.3)
GMFCS ≥ 3	875	0.9 (0.4, 2.2)	1.3 (0.4, 3.9)	4.0 (1.7, 9.6)	0.6 (0.2, 1.6)	0.7 (0.2, 2.3)	2.1 (0.8, 5.9)
Peds quality of life < 70	859	1.4 (0.9, 2.1)	2.5 (1.5, 4.1)	2.7 (1.6, 4.4)	1.1 (0.7, 1.8)	1.8 (1.0, 3.2)	2.0 (1.1, 3.5)

Abbreviations: ADHD, attention deficit hyperactivity disorder; ASD, autism spectrum disorder; GMFCS, Gross Motor Function Classification System; IQ, intelligence quotient; OR, odds ratio; ROP, retinopathy of prematurity; SRS, Social Responsiveness Scale (scores ≥ 65 indicates more social challenges).

Notes: Referent group is infants without ROP ( *n* = 223). Data are odds ratios (95% confidence intervals in parentheses).

a Multivariable analyses adjusted for maternal age, eligibility for government-subsidized health insurance, maternal years of formal education, marital status (married vs. unmarried), duration of mechanical ventilation, gestational age, any early or late sepsis, and surgical necrotizing enterocolitis (Penrose drain, exploratory laparotomy, or both).

b Sample size inadequate for estimation of odds ratio.

### Relationship of Eye Disorders/Vision Problems with Outcomes at 10 Years

Study participants are classified according to the most severe vision or eye problem using the following hierarchy: none, corrective lenses, strabismus not requiring surgery, strabismus requiring surgery, blind in one eye, or blind in both eyes ( Table 6 ).

**Table 6:** Associations between any vision/eye problem and outcomes at 10 years

Developmental outcome at 10 y	No vision/eye problem *N* = 474	Corrective lenses *n* = 226	Strabismus, no surgery *N* = 69	Strabismus, surgery *N* = 58	Blind, one eye *N* = 28	Blind, both eyes *N* = 34	*p* -Value
Cognitive impairment	94 (20.1%)	62 (27.7%)	7 (10.4%)	18 (31.0%)	12 (46.2%)	21 (67.7%)	<0.0001
Low score Word reading	44 (9.6%)	21 (9.5%)	2 (3.1%)	7 (12.1%)	8 (30.8%)	15 (51.7%)	<0.0001
Low score numerical operations	56 (12.0%)	38 (17.0%)	3 (4.5%)	15 (25.9%)	10 (38.5%)	13 (44.8%)	<0.0001
Autism spectrum disorder	24 (5.2%)	13 (5.9%)	7 (10.3%)	5 (8.9%)	8 (30.8%)	4 (19.0%)	<0.0001
SRS ≥ 65 (among those with no ASD and IQ ≥ 70)	37 (8.3%)	25 (11.4%)	12 (18.2%)	9 (16.7%)	1 (4.3%)	3 (15.0%)	0.07
ADHD	70 (15.0%)	44 (19.7%)	12 (17.6%)	13 (22.4%)	5 (18.5%)	8 (26.7%)	0.3
Anxiety-parent	42 (9.0%)	27 (12.1%)	8 (11.8%)	7 (12.1%)	1 (3.7%)	7 (23.3%)	0.1
Depression-parent	17 (3.7%)	10 (4.5%)	3 (4.4%)	3 (5.2%)	1 (3.7%)	1 (3.3%)	0.99
Anxiety-teacher	26 (7.8%)	15 (9.0%)	0 (0.0%)	4 (9.5%)	1 (4.5%)	4 (18.2%)	0.1
Depression-teacher	17 (5.1%)	4 (2.4%)	2 (3.8%)	0 (0.0%)	1 (4.5%)	1 (4.5%)	0.5
Epilepsy	21 (4.4%)	16 (7.1%)	5 (7.2%)	8 (13.8%)	4 (14.3%)	12 (36.4%)	<0.0001
GMFCS ≥ 3	15 (3.2%)	11 (4.9%)	2 (2.9%)	2 (3.4%)	2 (7.1%)	12 (35.3%)	<0.0001
Peds quality of life < 70	78 (16.7%)	63 (28.3%)	18 (26.1%)	25 (43.1%)	10 (37.0%)	20 (66.7%)	<0.0001

Abbreviations: ADHD, attention deficit hyperactivity disorder; ASD, autism spectrum disorder; GMFCS, Gross Motor Function Classification System; IQ, intelligence quotient; SRS, Social Responsiveness Scale (higher scores indicate more social challenges).

Notes: Study participants are classified according to the most severe vision/eye problem based on the following hierarchy: none/corrective lenses/strabismus not requiring surgery/strabismus requiring surgery/blind in one eye/blind in both eyes. *p* -Values are for chi-square test for difference in proportions within rows.

Overall, the percentage of adverse cognitive, behavioral, and neurological outcomes was similar in groups with current eye or vision problems as compared with groups without current eye or vision problems, unless the individual was blind in one or both eyes ( Table 7 ).

**Table 7:** Associations between any vision/eye problem and outcomes at 10 years

Developmental outcome at 10 y	Eyeglasses only *N* = 226	Strabismus, no surgery *N* = 69	Strabismus, surgery *N* = 58	Blind, one eye *N* = 28	Blind, both eyes *N* = 34
Cognitive impairment	1.5 (1.1, 2.2)	0.5 (0.2, 1.1)	1.8 (1.00, 3.3)	3.4 (1.5, 7.62)	8.4 (3.8, 18.3)
Low score word reading	1.0 (0.6, 1.7)	0.3 (0.1, 1.3)	1.3 (0.6, 3.0)	4.2 (1.7, 10.2)	10.1 (4.6, 22.3)
Low score numerical operations	1.5 (1.0, 2.4)	0.3 (0.1, 1.1)	2.6 (1.3, 4.9)	4.6 (2.0, 10.6)	5.9 (2.7, 13.0)
Autism spectrum disorder	1.5 (0.9, 2.5)	2.5 (1.2, 5.0)	2.2 (1.0, 4.8)	0.4 (0.1, 3.3)	1.1 (0.3, 3.9)
SRS > 65 (among those with no ASD and IQ ≥ 70)	1.1 (0.6, 2.3)	2.1 (0.9, 5.1)	1.8 (0.7, 4.9)	8.2 (3.2, 20.6)	4.3 (1.4, 13.8)
ADHD	1.4 (0.9, 2.1)	1.2 (0.6, 2.4)	1.6 (0.8, 3.2)	1.3 (0.5, 3.5)	2.1 (0.9, 4.8)
Anxiety-parent	1.4 (0.8, 2.3)	1.3 (0.6, 3.0)	1.4 (0.6, 3.2)	0.4 (0.1, 2.9)	3.1 (1.2, 7.6)
Depression-parent	1.2 (0.6, 2.8)	1.2 (0.4, 4.3)	1.4 (0.4, 5.1)	1.0 (0.1, 7.9)	0.9 (0.1, 7.1)
Anxiety-teacher	1.2 (0.6, 2.3)	a	1.3 (0.4, 3.8)	0.6 (0.1, 4.4)	2.6 (0.8, 8.4)
Depression-teacher	0.5 (0.2, 1.4)	0.7 (0.2, 3.3)	a	0.9 (0.1, 7.0)	0.9 (0.1, 7.0)
Epilepsy	1.6 (0.8, 3.2)	1.7 (0.6, 4.6)	3.5 (1.5, 8.2)	3.6 (1.1, 11.3)	12.3 (5.4, 28.4)
GMFCS ≥ 3	1.6 (0.7, 3.5)	0.9 (0.2, 4.1)	1.1 (0.2, 4.9)	2.4 (0.5, 10.8)	16.7 (7.0, 39.9)
Peds quality of life < 70	2.0 (1.3, 2.9)	1.8 (1.0, 3.2)	3.8 (2.1, 6.7)	2.9 (1.2, 6.6)	9.9 (4.5, 22.1)

Abbreviations: ADHD, attention deficit hyperactivity disorder; ASD, autism spectrum disorder; GMFCS, Gross Motor Function Classification System; IQ, intelligence quotient; SRS, Social Responsiveness Scale (higher scores indicate more social challenges).

Notes: Referent group is infants with no vision or eye problems ( *n* = 474). Data are unadjusted odds ratios (95% confidence intervals in parentheses).

a Sample size inadequate for estimation of odds ratio.

Table 8 (aORs) summarizes associations between eye problems and adverse outcomes at 10 years. Children who required corrective lenses but did not have strabismus or blindness (25.4% of participants) were more likely to have cognitive impairment and had lower scores for quality of life than those with no eye problems. Children who had strabismus but did not require surgery and were not blind (7.8% of participants) had higher odds of ASD. Children who had strabismus and required surgery (6.5% of participants) for strabismus were more likely to have lower scores for numeric operations, epilepsy, and lower scores for QoL. Lastly, children with blindness in one or both eyes (7% of participants) were more likely to have cognitive impairment, low academic achievement test scores for numeric operations and word reading, low social responsiveness, epilepsy, and low QoL.

**Table 8:** Associations between any vision/eye problem and outcomes at 10 years a

Developmental outcome at 10 y	Eyeglasses only *N* = 226	Strabismus, no surgery *N* = 69	Strabismus, surgery *N* = 58	Blind, one eye *N* = 28	Blind, both eyes *N* = 34
Cognitive impairment	1.3 (0.9, 2.0)	0.4 (0.1, 0.9)	1.6 (0.8, 3.1)	1.9 (0.8, 4.9)	6.3 (2.6, 15.2)
Low score word reading	0.8 (0.5, 1.5)	0.3 (0.1, 1.3)	1.0 (0.4, 2.6)	2.3 (0.8, 6.7)	8.2 (3.2, 21.0)
Low score numerical operations	1.4 (0.9, 2.4)	0.4 (0.1, 1.2)	2.6 (1.2, 5.3)	3.2 (1.2, 8.6)	4.7 (1.9, 11.9)
SRS > 65 (among those with no ASD and IQ ≥ 70)	1.2 (0.7, 2.2)	2.9 (1.3, 6.4)	2.3 (1.0, 5.4)	0.5 (0.1, 4.0)	2.5 (0.6, 9.6)
Autism spectrum disorder	1.1 (0.5, 2.3)	2.1 (0.8, 5.3)	1.2 (0.4, 3.4)	3.8 (1.2, 12.2)	2.6 (0.7, 9.0)
ADHD	1.3 (0.8, 2.1)	1.2 (0.6, 2.5)	1.3 (0.6, 2.7)	1.3 (0.4, 3.8)	1.7 (0.7, 4.4)
Anxiety-parent	1.2 (0.7, 2.1)	1.2 (0.5, 2.9)	1.3 (0.5, 3.1)	0.4 (0.1, 3.5)	3.0 (1.1, 8.2)
Depression-parent	0.9 (0.4, 2.2)	1.4 (0.4, 5.2)	1.3 (0.3, 4.8)	1.1 (0.1, 9.1)	0.7 (0.1, 5.7)
Anxiety-teacher	1.2 (0.6, 2.5)	b	0.6 (0.1, 2.6)	0.6 (0.1, 5.0)	3.0 (0.8, 11.0)
Depression-teacher	0.5 (0.2, 1.7)	0.8 (0.2, 3.8)	b	1.8 (0.2, 17.0)	1.6 (0.2, 15.4)
Epilepsy	1.3 (0.6, 2.6)	1.5 (0.5, 4.5)	3.6 (1.4, 9.1)	3.8 (1.1, 13.5)	12.8 (4.5, 36.6)
GMFCS ≥ 3	1.7 (0.7, 4.1)	1.0 (0.21, 4.6)	1.1 (0.2, 5.3)	2.5 (0.5, 12.6)	16.7 (5.7, 48.5)
Peds quality of life < 70	1.9 (1.2, 2.9)	1.9 (1.0, 3.6)	3.4 (1.8, 6.4	1.7 (0.6, 4.4)	9.4 (3.9, 22.7)

Abbreviations: ADHD, attention deficit hyperactivity disorder; ASD, autism spectrum disorder; GMFCS, Gross Motor Function Classification System; IQ, intelligence quotient; SRS, Social Responsiveness Scale (higher scores indicate more social challenges).

Notes: Referent group is infants with no vision or eye problems ( *n* = 474). Data are adjusted a odds ratios (95% confidence intervals in parentheses).

a Multivariable analyses adjusted for sex, maternal age, eligibility for government-subsidized health insurance, maternal years of formal education, marital status (married vs. unmarried), duration of mechanical ventilation, gestational age, any early or late sepsis, and surgical necrotizing enterocolitis (Penrose drain, exploratory laparotomy, or both).

b Sample size inadequate for estimation of odds ratio.

## Discussion

In this cohort of children born before 28 completed weeks of gestation, increased severity of ROP was associated with an increased severity of vision or eye problems, worse scores on math achievement tests, higher prevalence of anxiety, as reported by the parent, and lower QoL, as reported by the parent when the child was 10 years old. Moreover, we found that a history of blindness in one or both eyes was associated with these same outcomes, as well as worse scores on assessments of cognitive functioning, reading ability, and social responsiveness. Among the multiple potential explanations for the association we observed between more severe ROP and worse neurodevelopmental outcome are a shared etiology, visual functioning not assessed in our study, such as visuospatial ability, and unmeasured confounding.

Among participants in the large CRYO-ROP study, poor vision after threshold ROP was associated with an increased risk for disability 6 as well as worse educational outcomes and social skills at age 8 years. 15 At age 10 years, parental perspectives on health status and health-related HRQL were described in 244 participants with severe (i.e., threshold) ROP and 102 participants who did not develop ROP. Children with severe ROP were more likely to have functional limitations in vision, hearing, speech, ambulation, dexterity, emotion, cognition, and pain and reduction in HRQL. Among children who developed threshold ROP, those with poor vision outcomes had lower HRQL scores than those with better vision outcomes. 37 Although ROP was associated with parent-reported low QoL outcomes, it might not be associated with QoL outcomes as perceived by study participants themselves, since very preterm adolescents or young adults view QoL outcomes more positively than parents' assessments. 38

Several other studies report an association between severe ROP and neurodevelopmental disabilities in children, 39 40 41 including cognitive impairment and delayed psychomotor development. 7 42 43 44 45 46 In contrast, but consistent with our findings, others have found no association between severe ROP and neurodevelopmental outcome at 12, 24, and 36 months of age, after adjusting for birth weight, male sex, IVH, and eligibility for public health insurance. 47 In another recent study of extremely preterm children, children born extremely preterm who had ROP stage ≤ 3 that regressed without intervention were no more likely to have adverse neurodevelopmental at 2 years corrected age than children without a history of ROP. By contrast, children who underwent an ophthalmologic intervention for ROP had worse neurodevelopmental outcomes at 2 years corrected age than did children born extremely preterm who did not undergo intervention for ROP. 48 Sources of variation across studies of the relationship of developmental outcomes to ROP include differences in years and locations of births, neonatal treatments and co-morbidities, outcome assessments, and control of confounding. In addition, educational services and neuroplasticity, manifesting as improvement in neurodevelopmental functions, 48 49 may attenuate associations between ROP, related vision deficits, and neurodevelopmental outcomes in middle childhood.

## Strengths and Limitations

Our study has several strengths, including its large multicenter sample, increasing precision of odds ratio estimates. Second, a broad range of outcomes, including neuromotor, neurocognitive, neurobehavioral, and QoL, were collected prospectively by examiners who were unaware of study participants' ROP status. Third, we selected infants based on gestational age, not birth weight, to minimize confounding due to factors related to growth restriction. One limitation of our study is the sample attrition between the time of NICU discharge and follow-up, which was addressed by multiple imputations. Another is the lack of information about visual acuity, which precluded our ability to investigate whether deficits in visual acuity mediate the association that we observed between ROP severity and QoL in middle childhood. Lack of a term-born control group limits some of the inferences that can be drawn. The inclusion of term-born control group might have allowed us to control for the drift in IQ over time (the Flynn effect). Finally, the infants in our study sample were born during the years 2002 to 2004 and were classified when using, a now outdated International Classification of Retinopathy of Prematurity classification system. However, the current classification retains much of the items of the one we used, i.e., stage. 50 Since anti-vascular endothelial growth factor (VEGF) therapy was not used in 2002 to 2004, our findings cannot be generalized to cohorts born after the advent of this therapy. Current evidence is inconclusive as to whether anti-VEGF therapy alters neurodevelopment. 51

## Conclusion

In conclusion, 10 years after birth, ELGANs who had severe ROP, as compared with neonates without this condition, were more likely to have adverse outcomes at 10 years of age, including low scores on an assessment of math achievement, anxiety disorder, as reported by the parent, and lower quality of life, as reported by the parent. Somewhat reassuring are the findings that, in general, cognitive and neuromotor outcomes were not associated with the severity of ROP. Our finding that blindness in one or both eyes was associated with multiple adverse outcomes in middle childhood suggests that efforts to improve vision function, along with other developmentally supportive interventions, among infants with severe ROP could improve long-term outcomes for these infants. Conversely, our findings support cautious optimism when counselling families of extremely preterm infants who have experienced severe ROP but do not develop severe visual impairment.

## Notes

Summary Among children born extremely preterm, increased severity of retinopathy of prematurity was associated with increased severity of vision or eye problems, worse scores on math achievement tests, higher prevalence of anxiety as reported by parents, and lower quality of life as reported by the parents when their child was 10 years old. A history of blindness in one or both eyes was associated with these same outcomes, as well as worse scores on assessments of cognitive function, reading ability, and social responsiveness.

## Notes

**What's Known on This Subject**
Retinopathy of prematurity, a highly prevalent condition among extremely preterm neonates, has been associated with suboptimal neurodevelopmental outcomes, but studies of outcomes after early childhood are limited.
**What This Study Adds**
This study suggests that increased severity of retinopathy of prematurity, especially when followed by blindness in one or both eyes, is associated with worse outcomes in middle childhood.
